# Multi-Omics Integration Reveals the Crucial Role of *Fusobacterium* in the Inflammatory Immune Microenvironment in Head and Neck Squamous Cell Carcinoma

**DOI:** 10.1128/spectrum.01068-22

**Published:** 2022-07-11

**Authors:** Han Qiao, Hui Li, Xianhui Wen, Xirong Tan, Chongzhe Yang, Na Liu

**Affiliations:** a Department of Experimental Research, State Key Laboratory of Oncology in South China; Collaborative Innovation Center of Cancer Medicine; Sun Yat-sen University Cancer Center, Sun Yat-sen University, Guangzhou, China; b Zhongshan School of Medicine, Sun Yat-sen University, Guangzhou, China; c Department of Geriatrics, National Key Clinic Specialty, Guangzhou First People’s Hospital, School of Medicine, South China University of Technology, Guangzhou, China; University of Florida

**Keywords:** tumor microbiome, TME, TCMA, *Fusobacterium*, HNSC

## Abstract

The tumor microbiome is believed to have a profound impact on tumor progression owing to its local colonization in the tumor microenvironment (TME). Using the Cancer Microbiome Atlas (TCMA), a database of curated, decontaminated microbial profiles for 3,689 oropharyngeal, esophageal, gastrointestinal, and colorectal tissue samples from 1,772 patients, we conducted a comprehensive multi-omics analysis to reveal microbial signatures among various cancers and the potential mechanisms involved in tumor progression of head and neck squamous cell carcinoma (HNSC). We found that compared with other cancer types, the tumor-resident microbiome of HNSC accounted for the highest bacterial abundance and strongest association with host TME signatures. *Fusobacterium* was found to be enriched in HNSC tissues, which was associated with an increased inflammatory effect and inferior prognosis. Moreover, we revealed that the microbiota-associated inflammatory TME was attributed to the competing endogenouse RNA (ceRNA) network and chromatin accessibility.

**IMPORTANCE** Studies on revealing the composition and potential mechanisms of the tumor microbiome are still at an initial stage. We uncovered the potential contribution of the tumor-resident microbiota on the immunosuppressive microenvironment in HNSC, which will provide a new perspective for tumor microbiome research and yield valuable insights into the clinical management of HNSC.

## INTRODUCTION

Historical accounts linking cancer with microbes have developed over millennia, and almost 16% of cancer cases worldwide can be attributed to infectious factors (1). Notably, 11 kinds of microbes have been recognized as “oncomicrobes” by the International Association of Cancer Registry (IACR), among which only Helicobacter pylori belongs to *Bacteria*, and H. pylori also contributes to the carcinogenesis of gastric cancer (2). Recently, additional tumor-associated bacteria have been gradually demonstrated along with the update of microbial research technologies. However, emerging evidence suggests that human cancer can be attributed not only to single pathogens but also to disorders in our symbiotic microbiome (3).

Dysbacteriosis is reported to be involved in tumor carcinogenesis and progression through various mechanisms, including mutagenesis and epigenetic and immune activity changes of the host (4, 5). Bacteroides fragilis produces genotoxin B. fragilis toxin (BFT) and causes the host DNA damage to initiate colorectal carcinogenesis (6); Fusobacterium nucleatum targets innate immune signaling and miRNA-mediated autophagy to result in chemoresistance of colorectal cancer (7). In addition, the bacterial metabolite butyrate has been identified as a histone deacetylation inhibitor, suggesting that bacteria may influence tumor progression by affecting chromatin accessibility (8). Interestingly, it has been highlighted that the gut microbiome can affect the response to anti-PD-1 immunotherapy, suggesting the potential of the gut microbiome as a marker for the early prediction of the treatment efficacy of immune checkpoint inhibitors (9–11).

Multiple tumors occur on the surfaces of the human body that communicate with the outside world, such as the skin, oropharynx, respiratory tract, digestive tract, and urinary tract. The “cross talk” between the microbiota of these areas and tumor progression, such as the relationship between gut microbiome and colorectal cancer, has been studied extensively (12). Recently, studies have revealed the existence of intratumoral microbiota with metabolic activity across several cancer types using a combination of imaging, sequencing, and cultivation strategies and germfree mouse models (13). The tumor-resident microbiota makes up an important part of the tumor microenvironment (TME), which is considered to affect tumorigenesis and progression on a more local scale via regulation of the immune pathway, metabolism, and other functions of the host (5).

The Cancer Microbiome Atlas (TCMA) is a database of curated, decontaminated microbial profiles for 3,689 oropharyngeal, esophageal, gastrointestinal, and colorectal tissues from 1,772 patients based on whole-exome sequencing (WXS) and whole-genome sequencing (WGS) data that provides a useful tool for further exploring the function of microbes within indicated tumor tissues (14). In this study, combined with the multi-omics data of matched samples in The Cancer Gnome Atlas (TCGA), we aimed to reveal the microbial composition differences across various cancer types and further explore the potential mechanism of microbiota-associated tumor progression in head and neck squamous cell carcinoma (HNSC).

## RESULTS

### Distinct microbial diversity and composition are exhibited within various cancer types.

**FIG 1:**
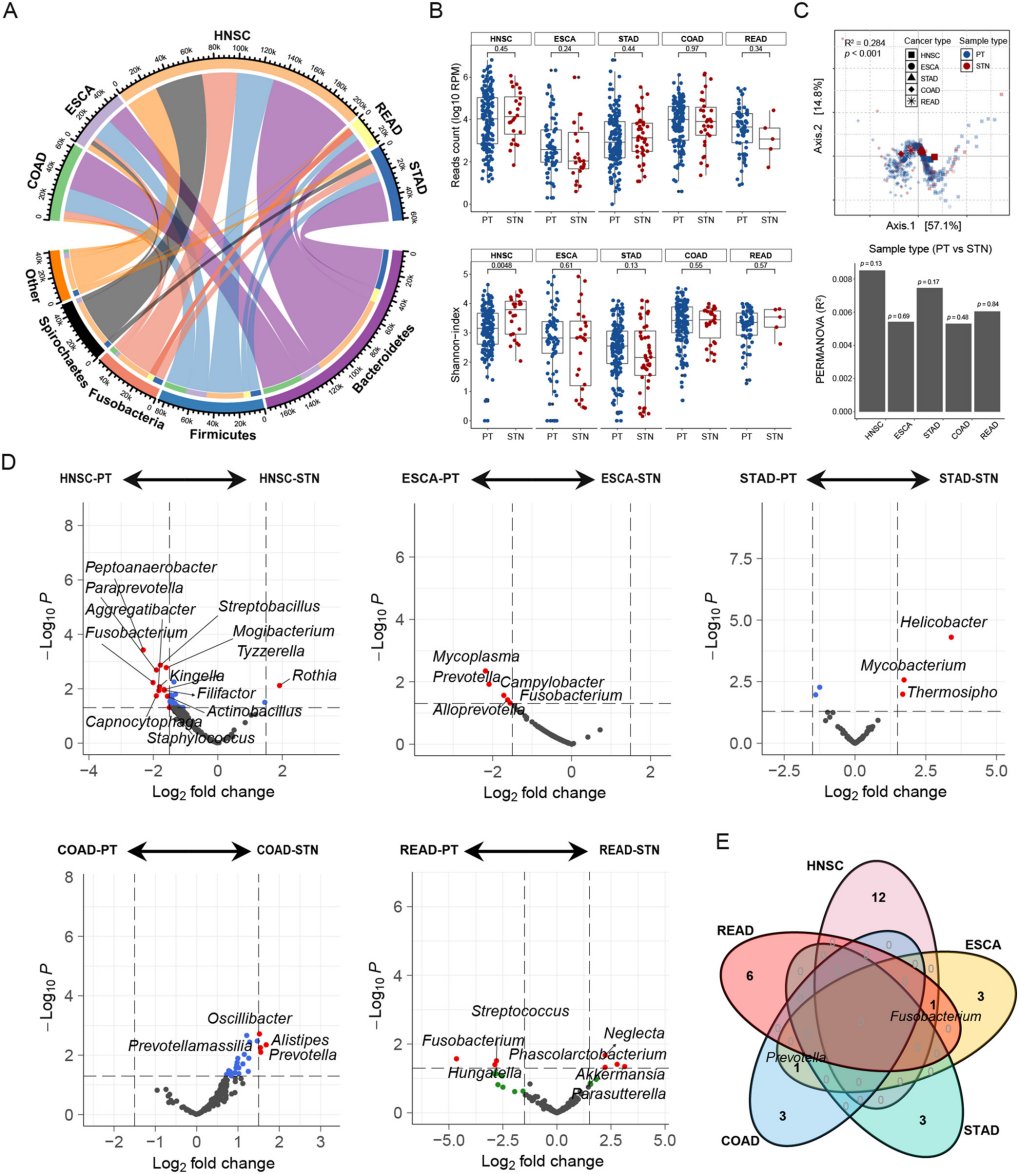
Distinct microbial diversity and composition exhibit within various cancer types. (A) The distribution of mean bacterial reads counts in head and neck squamous cell carcinoma (HNSC), esophageal carcinoma (ESCA), stomach adenocarcinoma (STAD), rectum adenocarcinoma (READ), and colon adenocarcinoma (COAD) tumor and normal tissues were analyzed. Representative bacteria with high abundance at the phylum level are presented. (B) The differences in the bacterial counts (top) and the alpha diversity (Shannon-index, bottom) were evaluated between primary tumor (PT) and solid tissue normal (STN) tissues. The comparison was performed with the Wilcoxon rank sum test. (C) PCoA was performed after calculating the Bray-Curtis distance based on CLR-transformed data, and the shapes in the scatterplot represent the five cancer types. PERMANOVA was used to calculate the significance of the bacterial beta diversity differences among various cancers (*P < *0.001, top), and the difference between the PT and STN tissues in each cancer type was calculated (bottom). (D) Volcano plot showing the differential genera based on the limma test between the PT and STN tissues in each cancer type. A fold change less than zero indicated that the genera were enriched in tumors; otherwise, they were enriched in normal tissues. (E) Venn diagram summarizing the occurrence profile of differentially abundant genera between the PT and STN tissues in various cancers.

We used the TCMA data set based on whole-genome sequencing (WGS), including the purified microbial compositions from oropharyngeal, esophageal, gastrointestinal, and colorectal tissues, to analyze the microbial characteristics in patients with HNSC (*n* = 196), esophageal carcinoma (ESCA; *n* = 94), stomach adenocarcinoma (STAD; *n* = 205), rectum adenocarcinoma (READ; *n* = 69), and colon adenocarcinoma (COAD; *n* = 183). The results showed that the bacteria within these tumor tissues belonged mainly to *Bacteroidetes*, *Firmicutes*, *Fusobacteria*, and *Spirochaetes* at the phylum level. Notably, HNSC tissues had more identified bacterial counts than other cancer types (Fig. 1A). No significant difference was observed in bacterial counts between primary tumor (PT) and solid tissue normal (STN) tissues of different cancer types. However, the alpha diversity in PT tissues of HNSC was lower than that of STN (Fig. 1B). Beta diversity showed significant differential clustering of microbiota in five cancer types, according to Bray-Curtis-based principal-coordinate analysis (PCoA) and permutational analysis of variance (PERMANOVA) (Fig. 1C).

Next, we used the limma test to identify differential species between PT and STN tissues. The results showed that genera such as *Fusobacterium*, *Peptoanaerobacter*, and *Staphylococcus* were enriched in HNSC, while genera such as *Prevotella* were enriched in ESCA and COAD. We observed that *Helicobacter* was enriched in the STN of STAD, while *Fusobacterium* was enriched in the PT of READ, supporting that they are known contributors to gastric tumorigenesis and colorectal cancer development, respectively (Fig. 1D). Venn diagrams exhibited the intersection of differentially abundant genera between the PT and STN in various cancer types (Fig. 1E).

### Cluster classifications of immune characteristics distinguish prognosis in HNSC.

**FIG 2:**
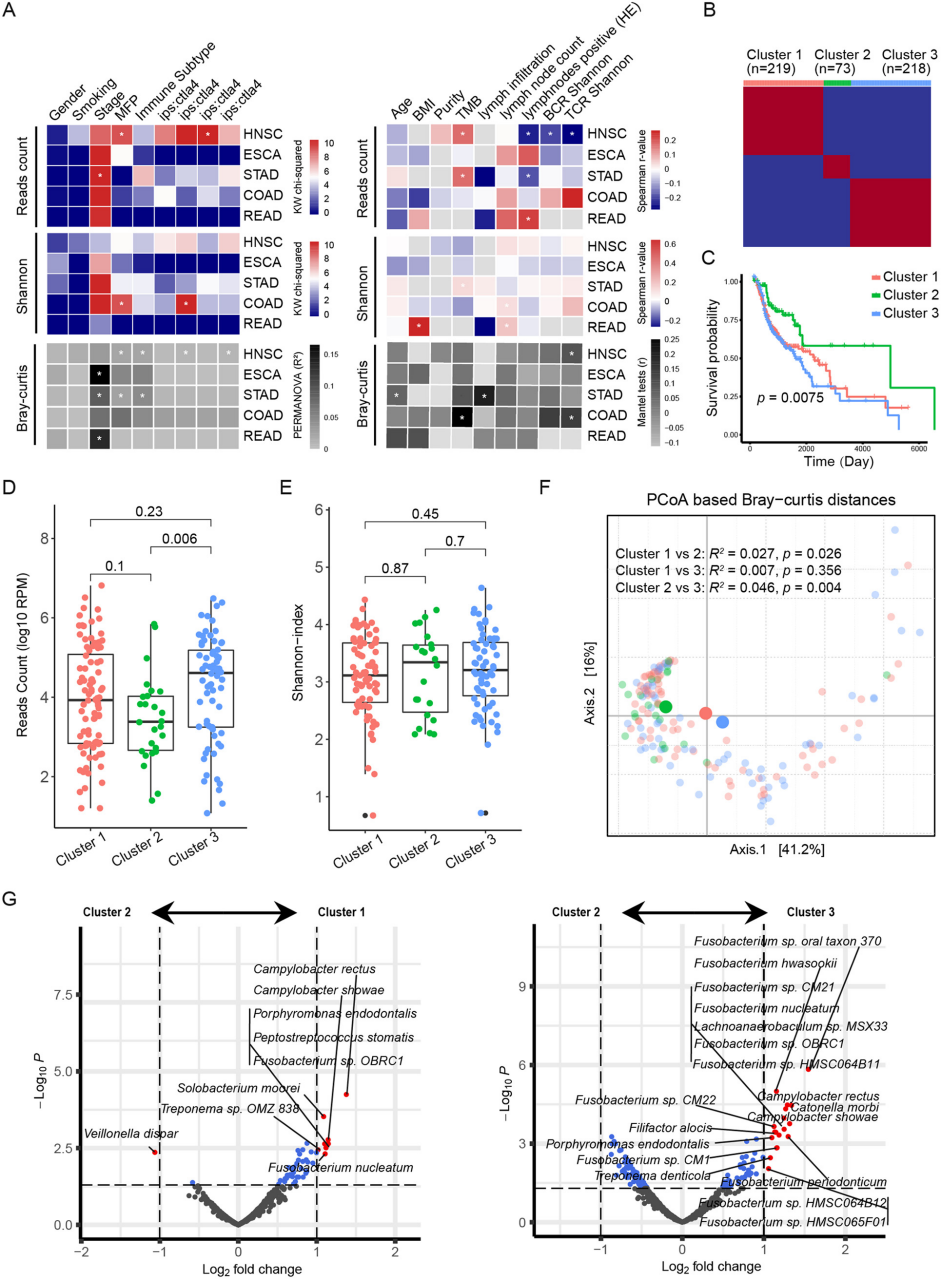
Cluster classifications of immune characteristics distinguish prognosis in HNSC. (A) The heatmap on the left panel shows the chi-square values of the Kruskal-Wallis test in comparing the difference in bacterial counts (top) and alpha diversity (Shannon-index, middle) among patient discrete variables, and the R2 value for PERMANOVA in evaluating the difference in beta diversity (Bray-Curtis distance). The heatmap on the right shows the Spearman r value between continuous variables and bacterial reads or alpha diversity, as well as the Mantel test r value of beta diversity based on the variables. * indicates statistical significance (*P < *0.05). (B) Based on TME signatures, HNSC patients were divided into three clusters by the NMF algorithm. (C) Kaplan-Meier curves of the three clusters. Significance was calculated using the log-rank test. (D and E) Differences in bacterial counts and alpha diversity among the three clusters. The comparison was performed with the Wilcoxon rank sum test. (F) PCoA was performed after calculating the Jaccard distance based on the CLR-transformed data, and the different colors in the scatterplot represent the three clusters. The significance of the microbial differences between the two groups was determined by PREMANOVA. (G) Volcano plot (based on the limma results) showing the differentially abundant bacteria between clusters 2 and 1 (left panel), as well as between clusters 2 and 3 (right panel).

vWe analyzed the clinical characteristics related to the microbial diversity and composition of tumor tissues, including gender, age, body mass index (BMI), smoking status, tumor purity, stage, tumor mutational burden (TMB) index, molecular functional portrait (MFP) signature, and immune characteristics defined by immune subtype, IPS score (defined as the number of tumor-associated immune cells with positive ctla4 staining/total number of tumor-associated immune cells), lymph node status, B-cell receptor (BCR) diversity, and T-cell receptor (TCR) diversity. The results of the Kruskal-Wallis test and Spearman analysis showed that the reads count of the tumor microbiota correlated with a variety of immune characteristics of HNSC, such as a negative correlation with lymph node status and BCR and TCR diversity and a positive correlation with IPS:ctla4 (Fig. 2A). Based on this, we speculated that the high abundance of tumor-resident bacteria in HNSC was related to an immunosuppressive microenvironment.

By analyzing the TME signatures of HNSC by GSVA (Table S1), HNSC patients were divided into three clusters by the NMF algorithm (Fig. 2B, Fig. S1, and Table S2). Survival analysis showed significant differences among these three clusters, and cluster 2 had a more favorable prognosis than the other two clusters (Fig. 2C). Next, we analyzed the bacterial reads count, diversity, and composition differences among three clusters. Cluster 2, with a favorable prognosis, had the lowest bacterial reads count, while cluster 3 had the highest (Fig. 2D). No significant difference was observed in alpha diversity among the three clusters (Fig. 2E). Additionally, clusters 1 and 3 had similar beta diversity, from which that of cluster 2 was significantly different (Fig. 2F). The limma test revealed that compared with that in cluster 2, HNSC in cluster 3 was enriched with multiple species of the genus *Fusobacterium* such as Fusobacterium nucleatum *and* Fusobacterium periodonticum (Fig. 2G).

### *Fusobacterium* correlates with an inflammatory TME in HNSC.

**FIG 3:**
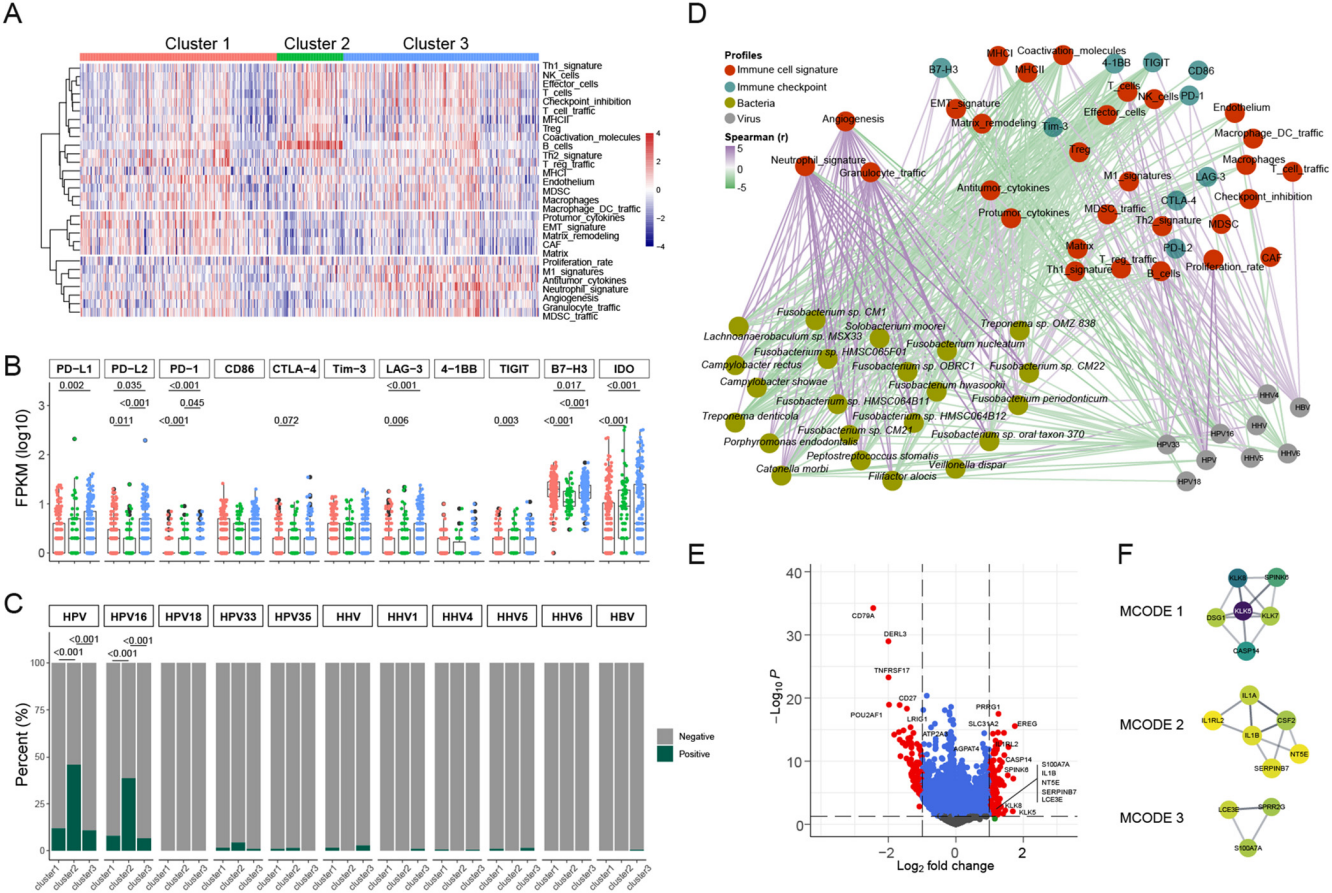
*Fusobacterium* correlates with an inflammatory TME in HNSC. (A) Heatmap showing the clustering results based on TME signatures. (B) The differential expression of immune checkpoints among the three clusters. The comparison between two groups was performed with the Wilcoxon rank sum test. (C) Differences in virus signatures among the three clusters, with HPV and HHV being the sum of their respective subtypes. The comparison between two groups was performed with the chi-squared test. (D) The cross-domain interaction networks across TME signatures, immune checkpoints, virus abundance, and bacteria (differential species of cluster 2 compared with cluster 1 or cluster 3) were calculated by Spearman test. The interaction network was visualized with Cytoscape software. The colors of nodes in the network represent different domains, and the green and purple edges indicate significant negative or positive correlations between nodes, respectively. (E) Volcano plot (based on the limma results) showing the differentially expressed genes (DEGs) between clusters 2 and 3. (F) DEGs in cluster 3 were applied to construct PPI networks with Cytoscape software, and the three highly interactive modules were recommended by the MCODE algorithm.

Based on the TME signatures, we further found that cluster 1 was characterized by a high level of epithelial-mesenchymal transition (EMT) signals, cluster 2 was mainly characterized by enrichment of B cells, NK cells, and other lymphocytes, and cluster 3 was dominated by inflammatory cells such as granulocytes (Fig. 3A). Compared with those in the other two clusters, high levels of checkpoints, including PD-L1, PD-L2, LAG-3, B7-H3, and IDO, were observed in cluster 3 (Fig. 3B). In addition, we revealed that HPV16 tended to be enriched in cluster 2 (Fig. 3C). By integrating an interaction network of TME features, checkpoints, virus abundance, and differential species, we found that the bacteria abundant in clusters 1 and 3 were mainly positively correlated with the characteristics of granulocytes, M1 signatures, angiogenesis, and B7-H3 and negatively correlated with the characteristics of MHCII, effector cells, TIGIT, and PD-1. It is notable that most viruses negatively interact with the differential species (Fig. 3D).

We explored whether cluster 3 had more prominent inflammatory infiltration characteristics in terms of gene expression and regulatory pathways than cluster 2. The limma analysis showed that enriched genes in cluster 3 included *EREG*, *PRRG1*, *SH2D5*, and *SLC31A2* (Fig. 3E and Table S3). Metascape analysis of these differential genes indicated that pathways enriched in cluster 3 included NF-κB, inflammatory, cell-cell adhesion, response to bacterium, etc. (Fig. S2A). We constructed a protein-protein interaction (PPI) network with enriched genes in cluster 3 (Fig. S2B), and minimal common oncology data elements (MCODE) analysis demonstrated that there were three core interaction sets comprising typical inflammatory factors (Fig. 3F). Our results suggest a crucial role of the microbiota in the immunoregulation of HNSC, and the enrichment of *Fusobacterium* is closely related to inflammatory characteristics.

### Mutation landscapes of microbiota-associated inflammatory genes in HNSC.

**FIG 4:**
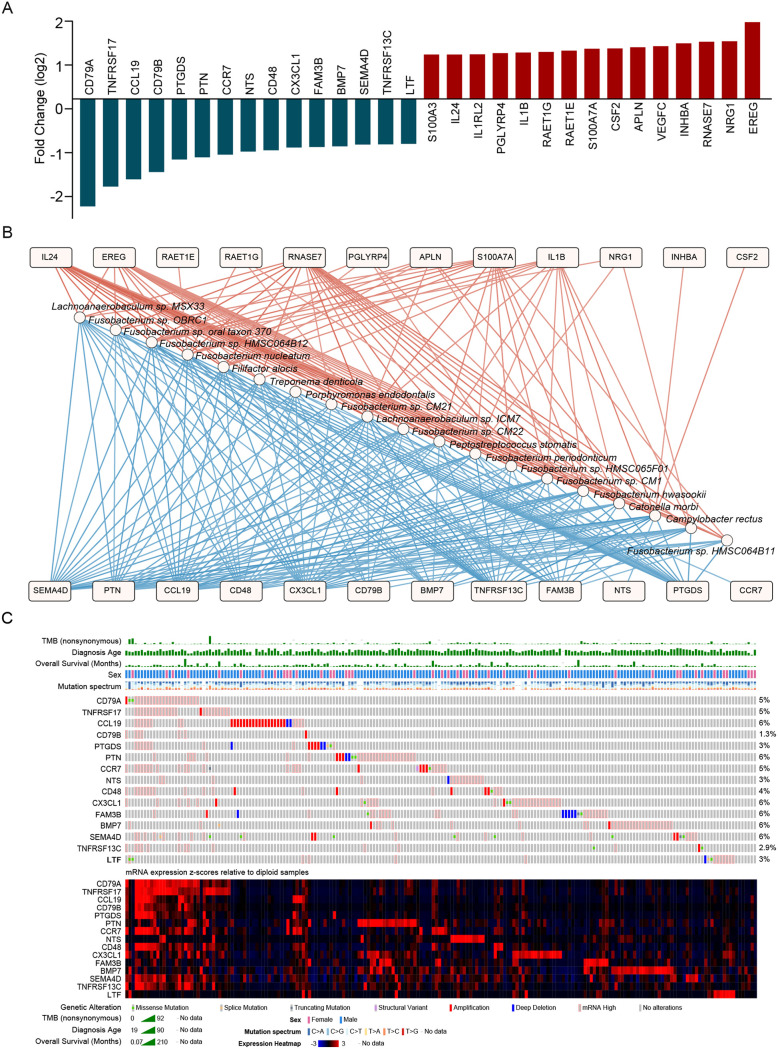
Mutation landscapes of microbiota-associated inflammatory genes in HNSC. (A) The significantly downregulated (blue) and upregulated (red) immune-related genes (based on ImmPort Portal) in cluster 3 compared to cluster 2. (B) Spearman association network of significantly differentially abundant bacteria (circle nodes) and differentially abundant immune genes (square nodes) between clusters 2 and 3. The red and blue edges connecting nodes represent significant positive or negative correlations. Only nodes with significant interactions (*P < *0.01, |r| > 0.3) were reported for this network. (C) The gene mutation and expression landscape of significantly downregulated immune-related genes in cluster 3 was analyzed in cBioPortal.

Based on the ImmPort data set (Table S4), we further revealed that immune genes, including EREG, IL1A/B, and INHBA, were enriched in cluster 3 compared to those in cluster 2, while CD79A, TNFRSF17, CCL19, etc. were decreased (Fig. 4A). The interaction network between the enriched species and differentially expressed genes from cluster 3 was constructed, and it was found that the species were positively correlated with enriched genes in cluster 3 and negatively correlated with the downregulated genes (Fig. 4B). Low gene expression due to microbiota-mediated gene mutations has been reported to play an important role in tumor development (5). We further constructed a gene mutation map of downregulated genes in cluster 3 based on the TCGA data set. However, we did not find any significant contribution of gene mutations to the expression of target genes, such as the presence of 5% abnormal gene expression in CD79A, which was not determined by gene mutation changes (Fig. 4C).

### Microbiota-associated inflammatory TME attributes to the ceRNA network and chromatin accessibility.

**FIG 5:**
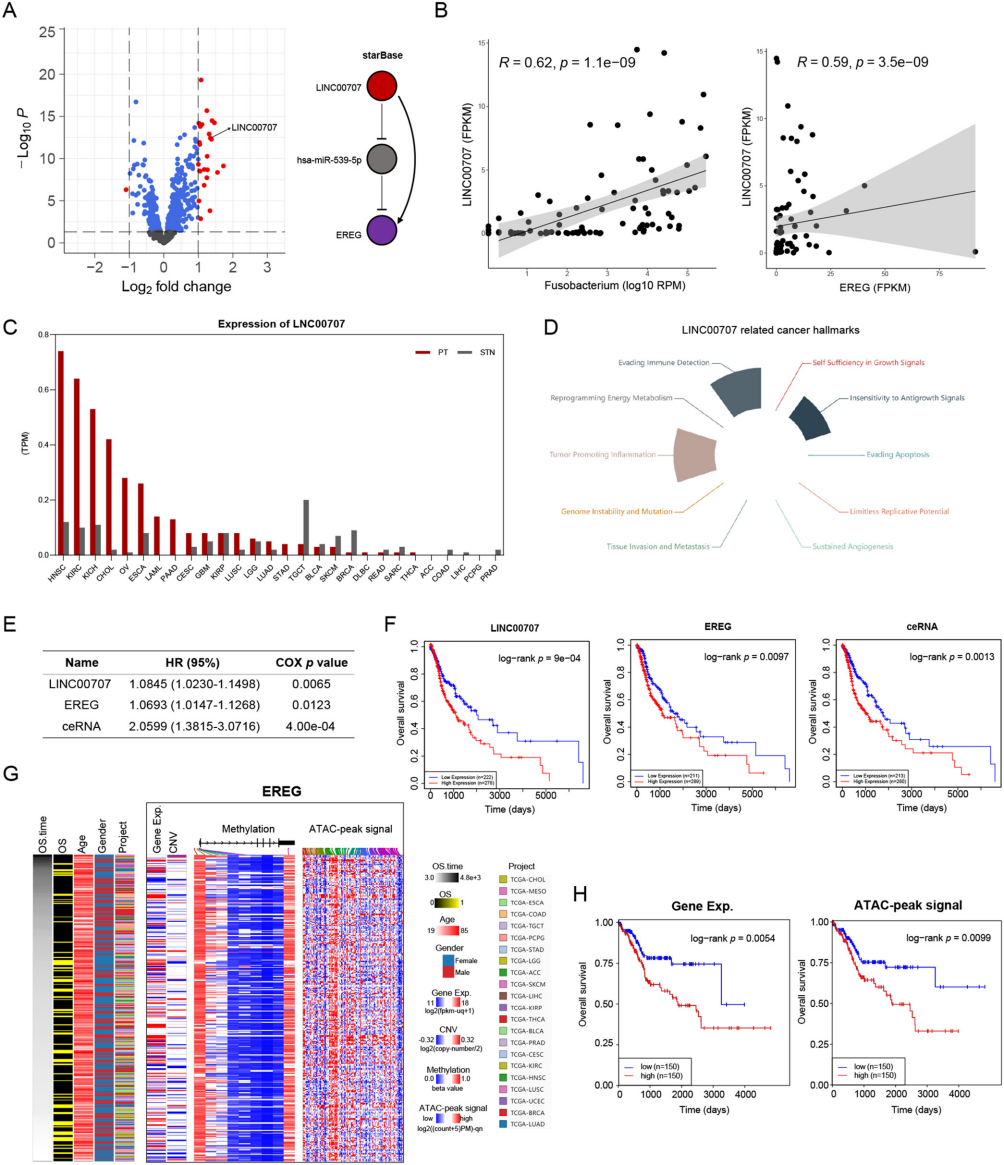
Microbiota-associated inflammatory TME attributes to the ceRNA network and chromatin accessibility. (A) Volcano plot showing the differentially expressed lncRNAs between clusters 2 and 3 analyzed with the R package GDCRNATools. Calculations based on the R starBase database identified the lncRNA-mediated ceRNA pathway (LINC00707, hsa-miR-539-5P, EREG). (B) Spearman correlations between the LINC00707 expression and *Fusobacterium* counts or EREG expression. (C) The bar plot shows the LINC00707 expression profile across the tumor and normal tissues in the TCGA pan-cancer data set. (D) s The ceRNA-related cancer hallmarks were identified with the LnCeCell database. (E and F) Cox regression analysis and survival curves for the ceRNA were performed with LnCeCell. (G and H) The multi-omics landscape for the EREG gene was completed at UCSC Xena, and survival analysis was performed with the median as the cutoff point.

We further utilized TCGA data sets, including long noncoding RNA (lncRNA), microRNA (miRNA), mRNA, DNA copy number variation (CNV), methylation, and assay of transposase accessible chromatin sequencing (ATAC-Seq), to clarify the possible mechanism of tumor-resident microbiota involved in regulating inflammatory genes differentially expressed between clusters 2 and 3 in HNSC. The differentially expressed lncRNAs and genes (DEGs), together with the miRNA and mRNA database, were used to calculate the ceRNA networks, and only one ceRNA network concerning the LINC00707-miR539-EREG axis was identified (Fig. 5A and Tables S5 to S6). The expression of LINC00707 was positively correlated with the bacterial count of *Fusobacterium* or EREG expression (Fig. 5B). Notably, the expression of LINC00707 in HNSC was higher than that in other cancer types, as well as normal tissues (Fig. 5C). Additionally, LINC00707 was mainly involved in hallmarks related to inflammation and immune escape (Fig. 5D). The Cox regression analysis showed that LINC00707, EREG, and ceRNA were significant risk factors for poor prognosis in HNSC patients (Fig. 5E). Survival analysis showed that patients with higher levels of LINC00707, EREG, and ceRNA had a poor overall survival in HNSC (Fig. 5F).

Subsequently, we analyzed the associations between EREG gene expression and DNA CNV, methylation, and ATAC-Seq using the pan-cancer atlas (Fig. 5G) and found that ATAC-Seq peak intensity of EREG was a significant factor affecting prognosis (Fig. 5H). In summary, multi-omics analysis demonstrates that epigenetic mechanisms such as the ceRNA network and chromatin accessibility play important roles in the interaction between the microbiota and the inflammatory TME.

## DISCUSSION

Dysbacteriosis has been linked to various health issues, including diabetes, inflammatory bowel disease, cancer, and other diseases. Dominated by tissue swabs and stool samples, the Human Microbiome Project and MetaHIT have immensely broadened our recognition and understanding of the human microbiome in terms of these physical sites (15, 16). Recently, based on intensely strict contamination control strategies, intratumoral bacteria have been identified and characterized with distinct compositions across seven cancer types, and they are correlated with the response to immunotherapy (17). Due to the crucial involvement of tumor-resident bacteria involved in cancer development and the difficulty of obtaining clinical samples, the researchers have designed a statistical model to analyze the prevalence of species across sample types from TCGA through a series of rigorous contamination treatments, thus proposing the TCMA data set (14). Based on this, combined with the multi-omics data of matched samples in the TCGA, we conducted an in-depth analysis to reveal microbial composition differences across cancer types and explore the potential mechanism of microbial-associated tumor progression in HNSC.

By comparing the microbial characteristics among different cancer types, we found that the microbiota of HNSC accounted for the highest bacteria reads count and had the strongest association with TME signatures. Limited studies have investigated the relationship between the human microbiota and HNSC carcinogenesis and progression, of which the oral microbiome has been mainly reported. The hypothesis that oral dysbacteriosis promotes HNSC development has been verified through antibiotic treatment and germfree HNSC mouse models (18). A prospective study has revealed that *Corynebacterium* and *Kingella* in the oral microbiome are associated with reduced HNSC risk (19). Recently, emerging studies have identified characteristic tumor microbiota in HNSC patients associated with poor prognosis (20, 21). Our analysis revealed that enrichment of *Fusobacterium* within tumors was collectively found in HNSC, ESCA, and READ, and it has been reported to act as an oncomicrobe. However, the impact and potential mechanism of *Fusobacterium* on tumor progression in HNSC remain unknown.

The opinion that chronic inflammation drives carcinogenesis has been widely demonstrated in various types of cancer. For example, hepatocellular carcinoma usually occurs in patients with chronic hepatitis (22), colorectal cancer may initiate with long-term untreated inflammatory bowel disease (IBD) (23, 24), and Helicobacter pylori infection-induced chronic gastritis is a well-known motivator of gastric cancer (25, 26). Microbes are closely related to host innate and adaptive immunity. However, the important role of the microbiota in tumorigenesis through immune regulation has just been recognized in recent years (27). We performed unsupervised clustering of immune cells in HNSC and identified three clusters with different prognoses. Cluster 3, characterized by a high level of inflammation such as granulocytes, had the worst prognosis, and the tumor tissues were enriched with *Fusobacterium*. This is consistent with a previous finding conducted in colorectal cancer, in which high Fusobacterium nucleatum abundance in the tumors correlates with M1 macrophage infiltration and inflammatory signals (28). Moreover, the study by Dou et al. also found that the abundance and composition of tumor microbiota in HNSC correlate with the inflammatory infiltration (20). Chronic inflammation has been linked to the reduced antitumor immune effect by enhancing the inhibitory activity of myeloid-derived suppressor cells (29), which may be the reason for the worst prognosis observed in cluster 3.

At present, studies regarding the microbiota on tumor progression have gradually stepped in the stage of causal evidence from brief associations. Strong experimental evidence suggests that human microbes initiate cancer through genotoxin-mediated mutagenesis, such as colibactin, cytolethal distending toxin, or Bacteroides fragilis toxin. In our present study, we found significant B-lymphocyte enrichment in cluster 2 and a decrease in relevant genes in clusters 3 and 1. However, we did not observe sufficient evidence that the bacterial community in HNSC downregulated these genes through mutagenesis. In colorectal cancer studies, Fusobacterium nucleatum is reported to promote metastasis by the miR-1322/CCL20 axis and M2 polarization (30) or chemoresistance by modulating autophagy through miR-18a* and miR-4802 (7). Multi-omics analysis in our study suggested that LINC00707/miR-539-5p may play an important role in regulating EREG, a target gene related to inflammation induced by *Fusobacterium*. In addition, we observed that the intensity of ATAT-Seq signals in EREG was correlated with poor prognosis of HNSC, suggesting that this may be another important regulatory mechanism in HNSC.

In conclusion, our study comprehensively characterized the composition of tumor-resident microbiome in HNSC and further uncovered its potential contribution to the immunosuppressive microenvironment and poor prognosis. Our findings will provide a new perspective for tumor microbiome research and yield valuable insights into the clinical management of HNSC.

## MATERIALS AND METHODS

### Data collection.

The decontaminated microbial composition data of HNSC, ESCA, STAD, READ, and COAD were downloaded from the TCMA database (https://doi.org/10.7924/r4rn36833) (14). The TMB index, IPS score, and TCR and BCR repertoire profiles of the five tumors were downloaded from MF Portrait (https://science.bostongene.com/tumor-portrait/) (31). The multiple virus signatures of HNSC were downloaded from the VirusScan pipeline, and the virus-positive or -negative criterion based on the cutoff of 100 reads was defined according to a previous report (32). Gene expression, miRNAs, DNA CNV, methylation, ATAC-seq peak-calling data, and clinical data of HNSC were downloaded from the UCSC Xena database (https://xenabrowser.net/datapages/).

### Microbiome data analysis.

The microbial abundance was evaluated using the PathSeq pipeline (33). The pipeline was based on prebuilt human and microbial reference genomes and the NCBI taxonomic database from the PathSeq resource package. Aligned bacterial sequencing reads were summarized at each taxonomic level for the available WGS data from the TCGA. Finally, the read counts for TCGA input bam files and PathSeq output bam files were calculated using SAMtools for reads per million (RPM) normalization, and total bacterial reads count values were normalized to total reads (per millions) for input bam files (14). Aggregated PathSeq results and associated metadata from all sequencing rums were deposited as phyloseq (v1.38.0) objects in R for downstream analysis (34). Next, the phyloseq file (named “physeq.WGS.solid.file.reads.rds”) based on the reads count was loaded, and the data set of HNSC (*n* = 196), ESCA (*n* = 94), STAD (*n* = 205), READ (*n* = 69), and COAD (*n* = 183) cohorts was used to calculate the Shannon-index (as alpha diversity) (35). The R “vegan” (v2.5.7) (36) package was used to calculate the Bray-Curtis distances (as beta diversity) (37) between samples based on centered log ratio (clt) transformed data, and the principal coordinates analysis (PCoA) (38) was also performed. PERMANOVA (39) or Mantel test (40) was used to evaluate the variation in microbial community related to discrete or continuous variables, respectively. The limma was also used to evaluate the differential species, with an adjusted *P* value of <0.05.

### TME signature and NMF-based clustering analysis.

The TME signature of the HNSC cohort (*n* = 510) was calculated by ssGSEA (31, 41). Then, the intensities were median-scaled for samples in the HNSC cohort. The scaled TME signatures of HNSC samples were used for NMF (v0.23.0) clustering, and the “lee” algorithm with iterations of 50 and ranks from 2 to 10 was performed. According to the indicators of cophenetic, dispersion, and silhouette indicators, three TME clusters were obtained. Next, the survival analyses among the three TME clusters in HNSC were performed using a log-rank test in the R “survival” (v3.2.13) package.

### DEG analysis.

To identify differentially expressed genes (DEGs) of the three TME clusters, we performed the differential analysis using the R package limma (v3.50.1) (42), which employs an empirical Bayesian approach to estimate gene expression changes using a moderated *t* test. The DEGs enriched in TME cluster 3 were determined by adjusted *P* value of <0.05, and the adjusted *P* values for multiple testing were calculated using the Benjamini-Hochberg correction.

### Network analysis.

The interaction network across TME features, checkpoints, virus abundance, and differential species was computed by Spearman rank correlation analysis. Potential interactions between DEGs enriched in TME cluster 3 were explored through the STRING database (https://string-db.org/) (43), and protein interaction networks were constructed to describe the regulatory relationships among these DEGs. The MCODE (44) algorithm was used to calculate the closely connected regions in the network. All of the copresence and mutually exclusive networks were analyzed using the Network analyzer tool of Cytoscape (v3.8.0) (45).

### ceRNA network analysis.

The standard R package GDCRNATools (v3.0) pipeline was used to analyze the lncRNA-mRNA-related ceRNA regulatory network between TME clusters 2 and 3 in the HNSC cohort. The differentially expressed lncRNA (*n* = 34) and DEGs (*n* = 23), together with the miRNA (*n* = 2,242) and mRNA (*n* = 56,602) databases, were used to calculate the ceRNA network.

### Web resource integration archives.

The DEGs enriched in TME cluster 3 were loaded into Metascape for functional enrichment analysis (http://metascape.org/gp/index.html) (46). The gene mutation and expression landscape of immune-related genes in HNSC atlas of TCGA was analyzed in cBioPortal (https://www.cbioportal.org/) (47). GEPIA2 was used to analyze the expression of LINC00707 in tumor and normal samples from TCGA data (http://gepia2.cancer-pku.cn/) (48). The survival analysis of LINC00707 and EREG in HNSC was performed in LnCeCell (http://bio-bigdata.hrbmu.edu.cn/LnCeCell/) (49), and the hallmarks of LINC00707 were also analyzed in LnCeCell. The multi-omics landscape for the RERG gene was completed at UCSC Xena with the GDC Pan-Cancer (PANCAN) database (*n* = 321), and survival analysis was performed with the median as the cutoff point.

### Statistical analysis.

All statistical tests between two groups were performed using the Wilcoxon rank sum test unless otherwise specified. Spearman analysis was performed to calculate linear associations between variables. Statistical tests of variance for microbial compositions were performed using PERMANOVA or the Mantel test. Statistical tests for survival analyses were performed using the log-rank test, and multivariate Cox regression analysis was used to test independent factors. For multiple tests, the false-discovery rate was calculated using the Benjamini-Hochberg method. All local analyses were performed in R, and the R scripts are available in the Git repositor (http://github.com/LIHUI92/TME_Microbiota).
