# Supplementary material for: Multi-Omics Integration Reveals the Crucial Role of Fusobacterium in the Inflammatory Immune Microenvironment in Head and Neck Squamous Cell Carcinoma
Source: Microbiol Spectr. 2022 Jul 11;10(4):e01068-22. doi: 10.1128/spectrum.01068-22 (PMC9431649; doi:10.1128/spectrum.01068-22)
Supplement: Supplemental file 1 — Supplemental material. Download spectrum.01068-22-s0001.pdf, PDF file, 0.4 MB [file spectrum.01068-22-s0001.pdf]

Supplemental Materials

FIG S1. NMF algorithm shows three optimal clusters.

FIG S2. Functional enrichment and PPI network of differential expressed genes.

Table S1. The tumor microenvironment signatures of HNSC was used for NMF clustering.

Table S2. HNSC patients were divided into three clusters by NMF.

Table S3. The result of limma analysis between cluster 2 and 3.

Table S4. Immunologically relevant list of genes in ImmPort Portal.

Table S5. The variables of lncRNA, DEGs, miRNA and mRNA were used to perform ceRNA network analysis.

Table S6. The ceRNA network with statistically significant was showed.

A

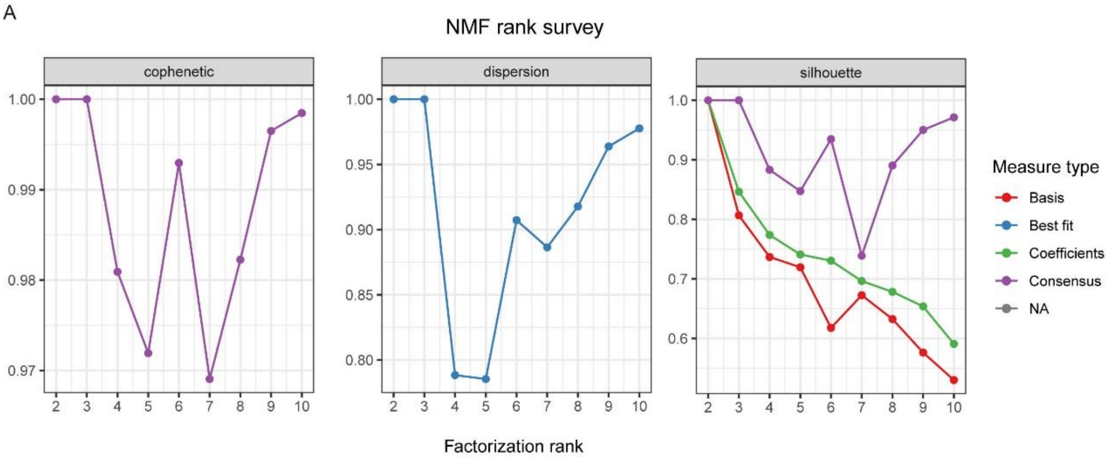

FIG S1 Based on the indicators of cophenetic, dispersion and silhouette, ranks of three was suggested as the best clusters

(A) According to the indicators of cophenetic, dispersion and silhouette, ranks of three was suggested as the best clusters.

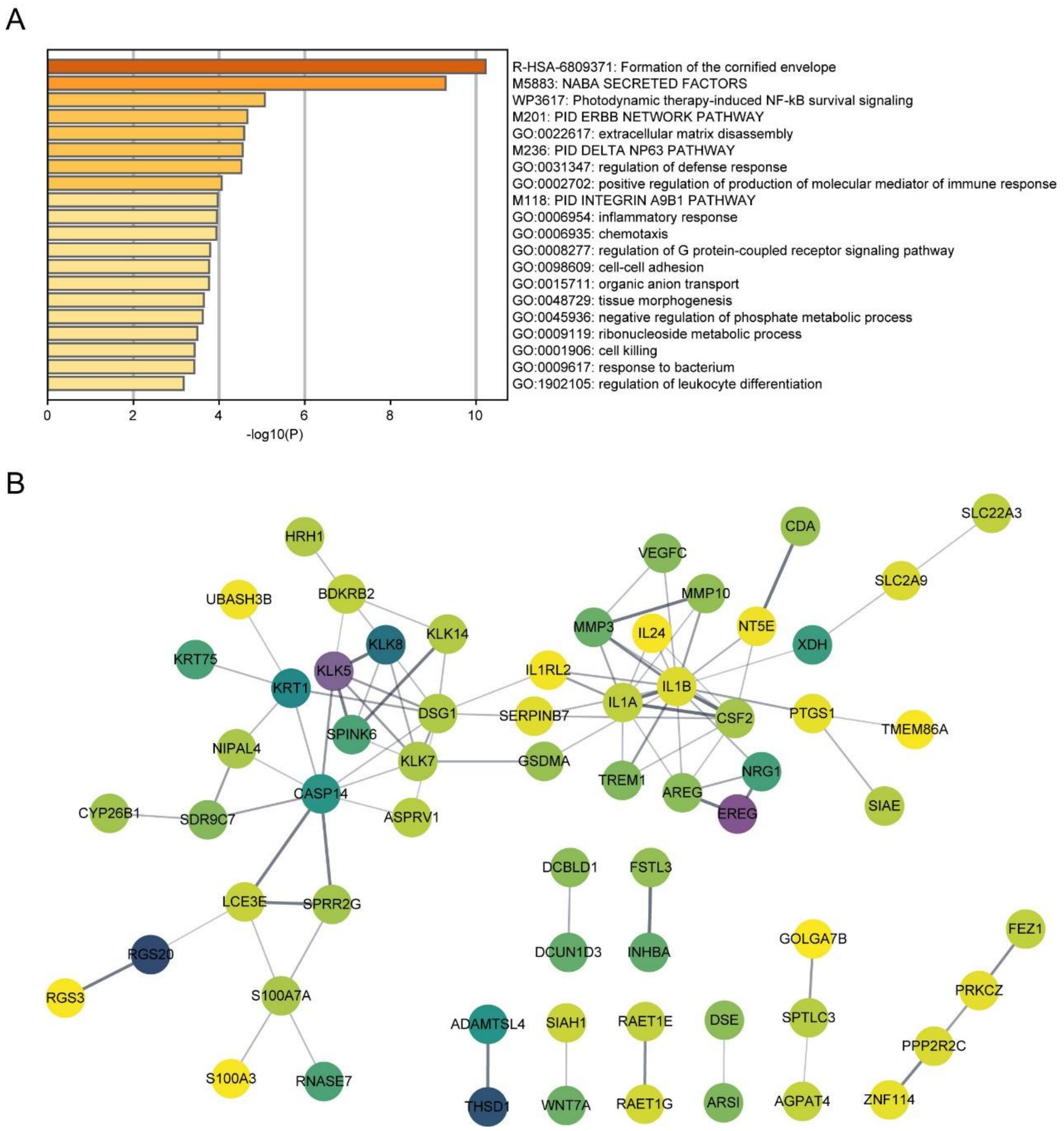

FIG S2 Functional enrichment and PPI network of differential expressed genes

(A) The overexpression genes in cluster 3 was loaded to Metascape (https://metascape.org/gp/index.html#/main/step1) for functional enrichment analysis based on GO, KEGG, Reactome and CORUM dataset.

(B) Potential interactions between DEGs were searched through the STRING database (https://string-db.org/), and protein interaction networks were constructed by Cytoscape.
